# Transcriptional profiling of the mutualistic bacterium *Vibrio fischeri* and an *hfq* mutant under modeled microgravity

**DOI:** 10.1038/s41526-018-0060-1

**Published:** 2018-12-18

**Authors:** Alexandrea A. Duscher, Ana Conesa, Mary Bishop, Madeline M. Vroom, Sergio D. Zubizarreta, Jamie S. Foster

**Affiliations:** 1 Department of Microbiology and Cell Science University of Florida, Space Life Science Lab, Merritt Island, FL 32953 USA; 2 Department of Microbiology and Cell Science Institute of Food and Agricultural Research, Genetics Institute, University of Florida, Gainesville, FL 32611 USA

## Abstract

For long-duration space missions, it is critical to maintain health-associated homeostasis between astronauts and their microbiome. To achieve this goal it is important to more fully understand the host–symbiont relationship under the physiological stress conditions of spaceflight. To address this issue we examined the impact of a spaceflight analog, low-shear-modeled microgravity (LSMMG), on the transcriptome of the mutualistic bacterium *Vibrio fischeri*. Cultures of *V. fischeri* and a mutant defective in the global regulator Hfq (∆*hfq*) were exposed to either LSMMG or gravity conditions for 12 h (exponential growth) and 24 h (stationary phase growth). Comparative transcriptomic analysis revealed few to no significant differentially expressed genes between gravity and the LSMMG conditions in the wild type or mutant *V. fischeri* at exponential or stationary phase. There was, however, a pronounced change in transcriptomic profiles during the transition between exponential and stationary phase growth in both *V. fischeri* cultures including an overall decrease in gene expression associated with translational activity and an increase in stress response. There were also several upregulated stress genes specific to the LSMMG condition during the transition to stationary phase growth. The ∆*hfq* mutants exhibited a distinctive transcriptome profile with a significant increase in transcripts associated with flagellar synthesis and transcriptional regulators under LSMMG conditions compared to gravity controls. These results indicate the loss of Hfq significantly influences gene expression under LSMMG conditions in a bacterial symbiont. Together, these results improve our understanding of the mechanisms by which microgravity alters the physiology of beneficial host-associated microbes.

## Introduction

All animals form beneficial relationships with microbes.^1^ The normal microbiota of animals is extremely important for maintaining almost every aspect of animal fitness including host development, behavior, and immune system homeostasis.^2,3^ Understanding how these beneficial microbes respond to their continually changing environments represents an important area in animal microbiome research. One particular environment that presents numerous physiological challenges to animals and their microbiomes is spaceflight.^4–8^ During spaceflight, the reduction in gravity, or microgravity, can have widespread health impacts to the host including bone loss, alterations to the genome, neurovestibular, and immune systems.^9–13^ In particular, animal immune systems are highly dysregulated and host–microbe interactions have now been shown to play a significant role in maintaining healthy immune function during spaceflight.^14^

In addition to physiological changes in human and animal hosts, microbes are also impacted by microgravity. Some microbes exhibit altered growth rates and cell densities grown under both natural and analog microgravity conditions.^15–20^ Although this is not a universal response as several taxa, including pathogenic *Streptococcus mutants* and *Salmonella enterica* Serovar Typhimurium, exhibit no changes to growth rates under modeled microgravity conditions.^21,22^ For many taxa, however, there is an increased growth rate under both natural and simulated microgravity conditions,^23,24^ which can be highly dependent on the growth media used.^19^ Although the precise mechanisms underlying the increased growth rate in certain taxa have not been fully elucidated, research has indicated that in some bacteria the lag phase of growth is shortened and the exponential growth phase is lengthened.^15^

Microbes also respond to changes in the mechanical and physical forces (e.g., low-shear) associated with microgravity by modifying their gene expression,^19,22,23,25–43^ secondary metabolism,^44,45^ biofilm formation,^26,28,34,40,43,46,47^ and pathogenesis.^48,49^ Many pathogenic microbes under microgravity conditions exhibit altered virulence,^21,39,47,50,51^ resistance to environmental stress and antibiotics,^27,29,34,35,37,40,42,48,52^ as well as increased survival in host macrophages.^21,39,42,49^ Previous studies have shown that these changes in virulence are environment-dependent and in some cases can be attenuated through media supplementation, such as inorganic phosphate.^51^ These same studies have also determined there are extensive changes in microbial gene expression both at the transcriptional and translational levels.

One key finding is that microgravity alters the expression of the global regulator Hfq, an RNA-binding protein that stabilizes an interaction between small RNAs (sRNAs) and their target message RNAs (mRNAs) to influence gene expression^53^ and has been found in about half of all known bacterial genomes.^54^ This protein has been implicated as an important mechanism involved in bacterial stress response, and therefore, may be especially important in microgravity conditions.^23,47,55^ Several studies have shown that the *hfq* gene is down-regulated in bacteria under natural and modeled microgravity conditions, including beneficial microbes.^23,47^

Although significant progress has been made in understanding microbial responses to microgravity, most of these studies have focused on pathogenic strains of microbes.^27,34,40,47,49^ The effects of microgravity and low shear fluid dynamics on mutualistic bacteria are relatively unknown. Two recent studies on gut-associated *Lactobacillus acidophilus* revealed relatively few transcriptional and physiological differences when cultures were grown under low-shear-modeled microgravity (LSMMG) conditions.^25,56^ For example, no transcriptomic or growth changes were observed when the cultivars were grown under anaerobic conditions,^56^ however, some increased acid stress resistance and antimicrobial activity was observed when grown under aerobic conditions,^25^ suggesting more investigations in how mutualistic bacteria respond to the stress of microgravity are needed.

In this study, we investigate the impact of LSMMG on the beneficial symbiont, *Vibrio fischeri*, which forms a simplified binary relationship with the bobtail squid *Euprymna scolopes*. *V. fischeri* colonizes the epithelial-lined crypt spaces of a specialized light organ in the squid and induces a series of rapid immunological and developmental changes in the host tissues.^57–60^ This type of colonization of host epithelial tissues represents the most common form of symbioses in animals.^61^ Previous research on the effects of modeled microgravity on the squid-vibrio system has identified several microgravity-induced phenotypes in the host tissue,^14,23,62,63^ however, the effects of LSMMG on the *V. fischeri* transcriptome has not been explored.

To address this issue, we examined the transcriptional response of *V. fischeri* cultures to LSMMG at both exponential (12 h) and stationary (24 h) growth phases. Additionally, the transcriptome of a *V. fischeri* mutant defective in *hfq* was also compared to determine the role of this transcriptional regulator in *V. fischeri* physiology under LSMMG conditions. Previous work has shown that the *hfq* gene is down regulated in *V. fischeri* during LSMMG and squid infected with ∆*hfq* mutants exhibited several altered developmental phenotypes.^23^ Together, this work helps elucidate the impact of microgravity and the importance of Hfq in a beneficial microbe. By understanding the effects that spaceflight has on beneficial microbes critical insight can be inferred into maintaining healthy astronaut microbiomes and decrease the potential health risks associated with the exploration of space.

## Results

### Overview of transcriptome analysis of *V. fischeri* cultivars under gravity and LSMMG conditions

RNA-seq was used to evaluate the transcriptional changes of wild type *V. fischeri* ES114 (WT) and a ∆*hfq* deletion mutant (KV7142) at two key time points during bacterial growth. Strains were grown aerobically in a rotary culture system using high aspect ratio vessels (HARVs) in both gravity and LSMMG positions and their transcriptomes were examined during exponential (12 h) and stationary (24 h) growth phases. Growth curves for all strains, including ∆*hfq* complementation mutants (KV148, KV149) are shown in Supplemental Fig. S1 and correlate with previously published studies.^23,62^ There was a statistically higher number of colony forming units per ml at 12 h in LSMMG conditions, but the growth curves suggest both the LSMMG-treated and gravity-treated cultures were in log phase growth. Three libraries were generated for each treatment (note: only two libraries were created for the ∆*hfq* gravity controls). An average of 11.19 million high-quality reads that consistently mapped (>95%) to *V. fischeri* ES114 genome were obtained for each treatment (Table 1). This level of sequencing depth in RNA-seq analyses has been shown to be effective in detecting the majority of significant changes to gene expression profiles in bacteria.^27,64^

**Table 1:** Overview of recovered transcriptome sequencing results from *V. fischeri* wild type (WT) and ∆*hfq* mutant exposed to low-shear-modeled microgravity (LSMMG) and gravity conditions

Time point (h)	Treatment	Strain	Total reads^a^ per treatment (million)^a^	Average reads per library (million)	Mapped reads (% mapped)
12	Gravity	WT	11.18	3.73	96.31
12	Gravity	∆*hfq*	7.20	3.60	96.20
12	LSMMG	WT	11.73	3.91	96.17
12	LSMMG	∆*hfq*	11.25	3.75	96.08
24	Gravity	WT	13.03	4.34	95.86
24	Gravity	∆*hfq*	8.40	4.20	96.49
24	LSMMG	WT	12.66	4.22	96.57
24	LSMMG	∆*hfq*	14.04	4.68	97.10

^a^High-quality reads were filtered using Trimmomatic default parameters

**Fig. 1:**
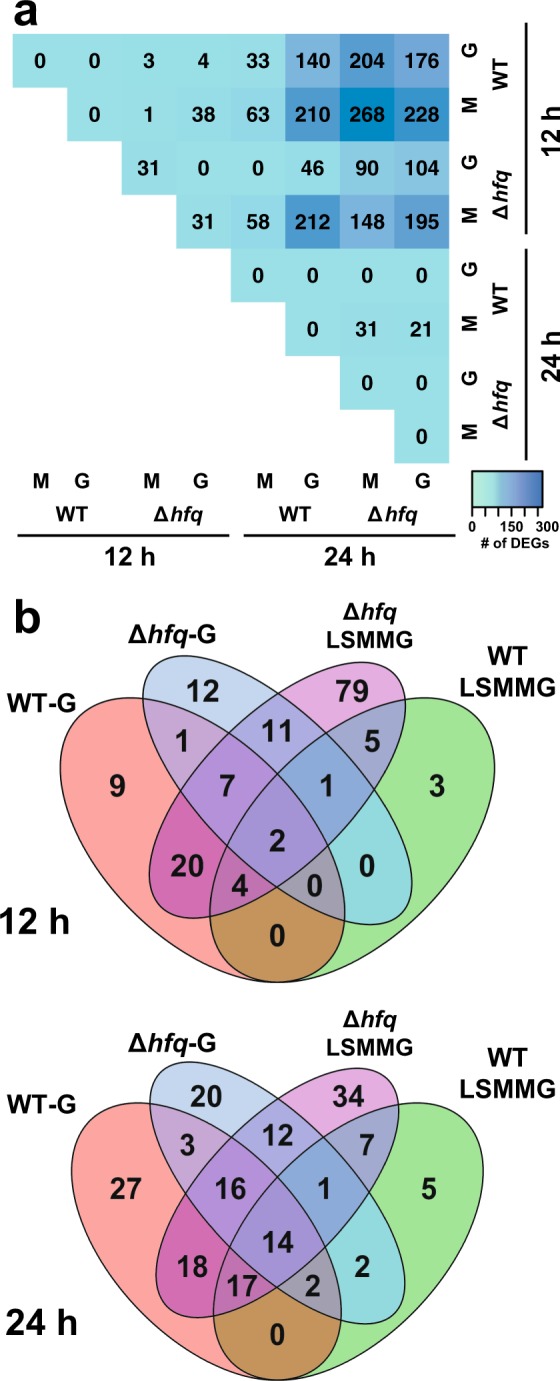
Overview of the differentially expressed genes associated to each of the eight transcriptomic comparisons in *Vibrio fischeri*. **a** Matrix of the significant differentially expressed genes (DEGs) in WT and ∆*hfq* mutants under LSMMG (M) and gravity (G) conditions with the colors reflecting the relative abundance. **b** Venn diagrams indicating the intersections of the significant DEGs shared between the different transcriptomic comparisons in this study at 12 and 24 h

**Fig. 2:**
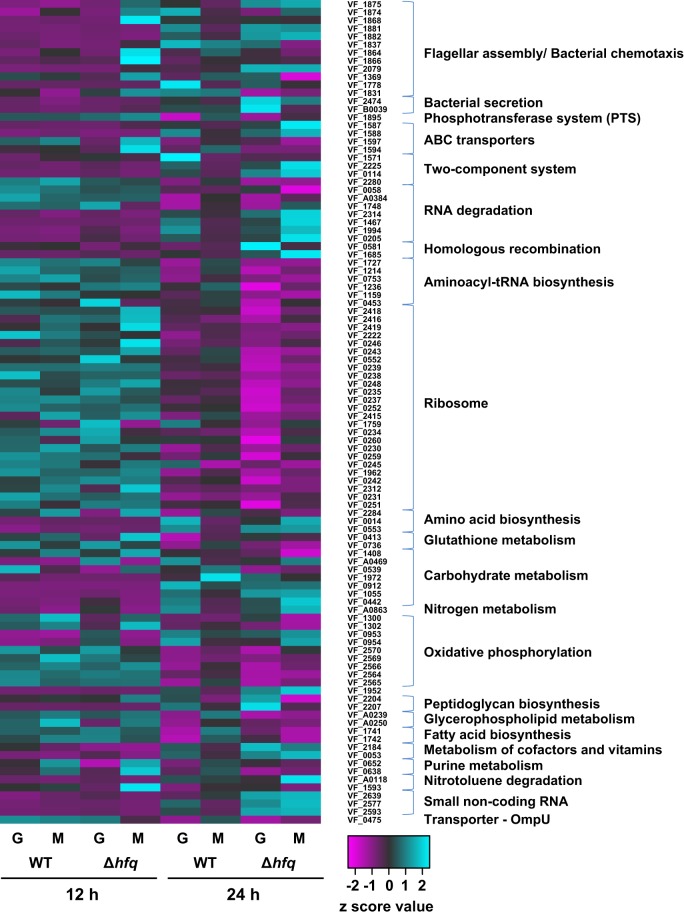
Heat map depicting the clustering patterns of the eight treatments by KEGG pathways associated with the proposed function of the *V. fischeri* genes at 12 and 24 h. Colors represent the differential abundance of individual genes listed by *V. fischeri* identification number (VF-ID) for both WT and ∆*hfq* mutants under low-shear modeled microgravity (M) and gravity (G) conditions

For control purposes, we first examined the transition between exponential and stationary phase in both WT and ∆*hfq* to ensure key metabolic transitions were being captured with the RNA-seq analyses in the HARV environment (Figs. 1 and 2). During growth under both LSMMG and gravity conditions the *V. fischeri* strains exhibited several typical responses of bacterial populations during stationary phase, including an overall down-regulation of genes associated with the translational apparatus, such as ribosomal proteins (e.g. *rpsB*, *rpsG*, *rpsL*, *rpsM*, *rplM*), tRNA synthases (e.g., *tyrS*, *leuS, lysS*), and translation factors (e.g., *tufAB, infC, miaA*) (Fig. 2; Supplemental Tables S1 and S2). In each treatment during stationary phase there was also an increase in the expression of several genes typically associated with stress responses, such as oxidative (e.g. VF_A0005, VF_A0335) and heat shock chaperones (e.g. *dnaK1, dnaK2*, *htpG*, *hslO*, *hslV*, *ibpA*, VF_1466) (Supplemental Tables S1–S3). These results are consistent with numerous studies indicating that in stationary phase bacteria become resistant to a wide range of environmental stresses^65,66^ and down-regulate their translational apparatus during nutrient limiting conditions.^67^ Together, the results indicate that the RNA-seq libraries were capturing the major transcriptional changes in *V. fischeri* during the different treatments.

### LSMMG-specific changes in *V. fischeri* transcriptome

Pairwise comparisons between the WT libraries revealed no significant differentially expressed genes (DEGs; adjusted *p*-value < 0.05) between the LSMMG and gravity treatments for WT at each of the time points tested, suggesting that the modeled microgravity environment had an overall minimal impact on the transcriptome of WT *V. fischeri* (Fig. 1). However, a comparison between the time points revealed five LSMMG-specific upregulated DEGs in both the WT and ∆*hfq* cultivars at 12 h when compared to 24 h (Fig. 1b; Supplemental Table S3). Two of these DEGs were associated with stress responses, including open-reading frame (ORF) VF_2561, whose gene product was annotated as a cold shock protein, and *yceD*, which encodes for a hypothetical protein that has been implicated in oxidative stress resistance in *Bacillus subtilis.*^68^

At 24 h there was an up-regulation of seven LSMMG-specific genes in both *V. fischeri* WT and ∆*hfq* strains when compared to 12 h libraries (Fig. 1b; Supplemental Table S3), several of which are known to be critical for stress resistance and microbial pathogenesis. For example, there was an up-regulation of *yghU*, which encodes for glutathione S-transferase and is essential for the detoxification of reactive oxygen species (ROS) in a wide range of taxa^69^ including several symbiotic taxa.^70,71^ There was also an increase in expression of *blc*, which encodes for the outer membrane lipoprotein lipocalin that is upregulated under high osmotic stress conditions in *Escherichia coli* and thought to play a role in antimicrobial resistance in several other bacteria.^72^ Additionally, there was an increase in expression of *zwf*, which encodes for glucose 6-phosphate dehydrogenase (G6PD), and has been shown to be required for virulence in *Salmonella* Typhimurium and protects against reactive oxygen and nitrogen species in both *S*. Typhimurium and *E. coli.*^73,74^ There was also up-regulation of *katA*, which encodes for the only periplasmic catalase present in the *V. fischeri* genome and is induced under oxidative stress conditions, as well as required for symbiosis competence in *V. fischeri.*^75^

### Differential gene expression changes in ∆*hfq* mutant under both gravity and LSMMG conditions during exponential phase

**Fig. 3:**
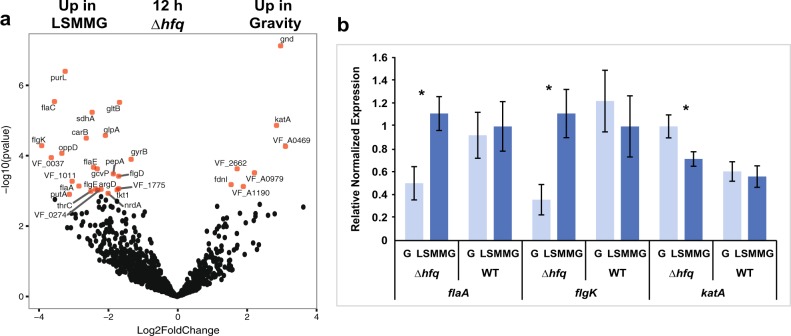
Differential gene expression between LSMMG and gravity (G) in a ∆*hfq* mutant at 12 h. **a** Volcano plot visualizing the global transcriptional changes within the mutant cells. All normalized transcripts were plotted and each point reflects one gene with those in red indicating significance (adjusted *p*-value < 0.05). Full details of the significant differentially expressed genes are listed in Supplemental Table S1. **b** Quantitative real-time PCR of selected genes compared under LSMMG and gravity conditions in WT and ∆*hfq* mutants. Genes selected were significant in the RNA-seq results and included genes encoding a flagellin structural protein (*flaA*), flagellar hook-associated protein (*flgK*), and catalase (*katA*). Asterisk indicates significance between comparisons and error bars reflect the standard error of the mean

In gravity conditions, there were few significant DEGs upregulated in the ∆*hfq* mutant compared to LSMMG at 12 h (Figs. 1 and 3a; Supplemental Table S1). One DEGs upregulated in gravity conditions was *gnd*, which encodes for 6-phosphogluconate dehydrogenase (6PGD), a key enzyme in the pentose phosphate pathway. The 6PGD enzyme produces NADPH, which provides the reducing power to several antioxidant proteins.^76^ Additionally, in the ∆*hfq* mutant, there was an increase in the expression of *katA* in gravity compared to LSMMG. The RNA-seq trends for *katA* were independently confirmed with qRT-PCR, although different transcript abundances were observed between the two methodologies for *katA* likely due to the differences in resolution between the approaches (Fig. 3b). At 12 h there were also three ORFs with unknown function upregulated in the ∆*hfq* gravity conditions (VF_2662, VF_A0979, and VF_A1190) (Supplemental Table S1).

Under LSMMG conditions, however, the ∆*hfq* mutant exhibited a pronounced change to its transcriptome compared to gravity controls at 12 h (Figs. 1a and 3a; Supplemental Tables S1 and S2). During exponential phase in the ∆*hfq* mutant there was an accumulation of transcripts that encode for several components of the tricarboxylic acid (TCA) cycle including succinate dehydrogenase (*sdhAB*), aconitate hydratase (*acnB*), succinyl-CoA synthetase (*sucCD*), fumarate hydratase (*fumB*), and fumarate reductase (*frdA*) (Fig. 3a; Supplemental Table S1), all of which have been shown to be repressed by the sRNA RyhB in other taxa.^77,78^ Hfq is required for the stability and pairing of the sRNA RhyB to mRNA.^78^ RhyB has been identified in the *V. fischeri* genome (VF_2578), however, it was not significantly differentially expressed in this study. Additionally, there was an enrichment of transcripts associated with fatty acid synthesis (e.g. *fabDFH*), which in *Salmonella* Typhimurium is dependent on the sRNA SmpP,^79^ as well as oligopeptide transport (e.g., *oppADF*), which is regulated by the Hfq-dependent small RNA GcvB in a number of taxa including several vibrios.^80^ Homologs to SmpP and GcvB have not yet been reported in *V. fischeri*.

In LSMMG conditions there was also an increase in transcripts associated with flagella synthesis in exponential phase including genes that encode for both structural (e.g. *flaACE*), basal body rod (e.g. *flgD*), and hook-associated (e.g. *flgEK*) proteins^81^ (Fig. 3a; Supplementary Tables S1 and S2). The differential expression of *flaA* and *flgK* were confirmed with qRT-PCR in the ∆*hfq* (Fig. 3b). Hfq has been associated with flagellar synthesis in a wide range of taxa, including both pathogenic (e.g., *Salmonella*) and mutualistic bacteria (e.g., *Sinorhizobium meliloti*), however, in most cases mutants defective in *hfq* exhibit a repression of flagellar synthesis genes and in some cases are non-motile.^82,83^

### Differential gene expression changes in ∆*hfq* mutant under both gravity and LSMMG conditions during stationary phase

**Fig. 4:**
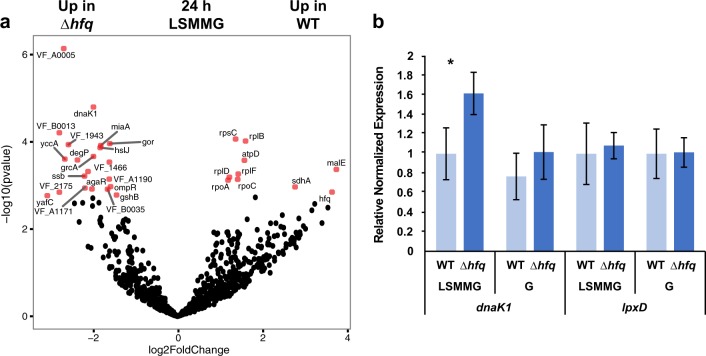
Differential gene expression between WT and ∆*hfq* mutant under LSMMG at 24 h. **a** Volcano plot visualizing the global transcriptional changes within the *V. fischeri* cells. All normalized transcripts were plotted and each point reflects one gene with those in red indicating significance (adjusted *p*-value < 0.05). Full details of the significant differentially expressed genes are listed in Supplemental Table S1. **b** Quantitative real-time PCR of selected genes compared under low-shear modeled microgravity (LSMMG) and gravity conditions in wild type (WT) and ∆*hfq* mutants. Genes selected included *dnaK*, which encodes for a stress-associated chaperone protein and *lpxD*, which assists in the biosynthesis of lipid A. Asterisk indicates significance between comparisons and error bars reflect the standard error of the mean

The ∆*hfq* mutants exhibited extensive transcriptional changes during stationary phase under both gravity and LSMMG conditions (Figs. 1, 2, and 4; Supplemental Tables S1 and S2). One pronounced characteristic of the ∆*hfq* transcriptomes was the up-regulation of numerous transcriptional regulators during stationary phase (Fig. 4; Supplemental Tables S1 and S2). In both gravity and LSMMG conditions there was increased expression of *agaR*, which encodes for a putative transcriptional repressor of N-acetyl galactosamine (GalNAc) transport and metabolism in a wide range of bacterial taxa;^84^ *iscR*, a transcriptional repressor of genes associated Fe–S cluster assembly proteins;^85^ and *yqhC*, whose gene product regulates aldehyde reductase.^86^

In gravity conditions, there was differential expression of VF_1401, which encodes for a Fis family transcriptional regulator, and c*ysB*, which belongs to the LysR family of regulators and is a global transcriptional activator of cysteine biosynthesis and sulfur metabolism.^87^ CysB is also the only known negative regulator of HslJ, a heatshock/heat-inducible outermembrane lipoprotein.^88^ The *hslJ* gene is upregulated in the ∆*hfq* mutant under both gravity and LSMMG in stationary phase (Fig. 4a; Supplemental Tables S1 and S2). Additionally, in gravity conditions, the ∆*hfq* mutant had increased expression of genes associated with the Type II section pathway (e.g. *gspD*, *mshQ2*) and several transport proteins (e.g. *ybhG*, *argT*, *hisP*) (Supplemental Tables S1 and S2).

Under LSMMG conditions, the genes of several different transcriptional regulators were upregulated during stationary phase. For example, VF_2037, which shares similarity to Cro/Ci family transcriptional regulators, was upregulated but has unknown function in *V. fischeri*. Additionally, *nrdR* was also upregulated and its gene product represses the ribonucleotide reductase production (i.e., *nrdHIEF*), bacterial chemotaxis, and more recently has been shown to inhibit cell adhesion to epithelial cells in *E. coli.*^89,90^ In addition to transcriptional regulators, there was also a differential expression of genes associated with modifications to the outer membrane in ∆*hfq* under LSMMG (Fig. 4a; Supplemental Tables S1 and S2). For example, there was increased expression of the gene *slp*, which encodes for an outer membrane lipoprotein associated with stress responses during stationary phase and is typically repressed by the Hfq-dependent sRNA GcvB.^91^ There was also an increased expression of *skp*, a periplasmic chaperone protein that is associated with the RpoE regulon and is involved in the folding of intermediates of outer membrane proteins.^92^ Interestingly, *rpoE* transcription was down-regulated at 24 h compared to 12 h in the ∆*hfq* mutants irrespective of the gravity or LSMMG treatment (Supplemental Table S1).

## Discussion

To prepare for long-duration space travel it is essential to have a comprehensive understanding of the impact that spaceflight has on the physiology of host-associated microbiomes to promote and maintain astronaut health. There has been an extensive focus on the effects of spaceflight and simulated microgravity environments on bacterial pathogens,^19,21,22,27,32,34,42,43,47,52^ however, only a few studies have begun to examine the impact on beneficial microbes that promote the health of the host organism.^14,23,25,56,62^ In this study, we expand on this recent work and examine the effects of an LSMMG environment on the transcriptome of the beneficial symbiont *V. fischeri*, which forms a mutualistic association with the bobtail squid *E. scolopes*, and is critical for the host’s normal development. The results of this study suggest that there were few transcriptional changes in the WT *V. fischeri* under LSMMG and that most changes in the bacterium were attributed to the growth phase transition between exponential and stationary phase. Additionally, RNA-seq analyses revealed that mutants defective in the global regulator Hfq exhibited a pronounced change in transcriptional profiles under LSMMG, providing new insight into the role this regulator plays in the symbiotic *V. fischeri* under analog microgravity conditions.

Previous studies have shown that *V. fischeri* exhibits an altered growth response in simulated microgravity conditions, with cultures reaching higher cell densities compared to gravity controls.^23^ This altered growth response under LSMMG has been observed in many, but not all, taxa^18,22,32,42^ and is thought to reflect the selected growth medium. The nutritional micro-environment of the cells in LSMMG has been shown to significantly impact microbial physiology.^18,19^ For example, under low phosphate conditions some microbes, such as *Salmonella* Typhimurium, exhibit increased virulence.^51^ Despite the change in growth phenotype in *V. fischeri* under LSMMG conditions, no significant DEGs were observed when the transcriptomes of 12 h LSMMG-treated WT cells were compared to 12 h gravity controls, even under the low phosphate conditions of SWT media (Fig. 1). Similar results were observed when 24 h LSMMG-treated libraries were compared to 24 h gravity-treated libraries, suggesting that modeled microgravity itself does not significantly alter transcription within *V. fischeri* compared to gravity controls. These results are comparable to several recent studies on the effects of LSMMG on the probiotic strain *L. acidophilus.*^25,56^ Under the anaerobic conditions, transcriptomes of the *L. acidophilus* cultivar showed no DEGs in LSMMG when compared to gravity controls at stationary phase.^56^ As both *L. acidophilus* and *V. fischeri* typically form associations with host epithelium and are regularly exposed to low shear conditions in their natural environments, the modeled microgravity environment does not likely impose a significant stress for these taxa. Additionally, recent studies have shown that under LSMMG conditions *V. fischeri* exhibits no delay in colonizing host tissues^62^ and that during spaceflight *V. fischeri* reached the same colonization densities as under gravity controls.^14^ Together, these results suggest that microgravity conditions do not negatively impact *V. fischeri*.

Although the overall transcriptional response in *V. fischeri* was typical of the normal transition to stationary phase, there were several stress-associated genes differentially expressed under LSMMG conditions in both the WT and ∆*hfq* mutant. Of the DEGs differentially upregulated in LSMMG during the transition to stationary phase, three of the observed genes (i.e., *yceD*, *yghU*, and *katA*) are associated with stress responses and have been observed in *E. coli* K12 under modeled microgravity conditions.^38^ In *E. coli* these genes are associated with both oxidative and osmotic stress responses and may suggest that under LSMMG a small microenvironment of increased stress may occur around the *V. fischeri* cells. The formation of nutrient-depleted microenvironments has long been postulated under LSMMG conditions, which may simulate the genomic and physiological responses of cells as they transition to stationary phase.^15,38^ During exponential phase, *V. fischeri* cells are flagellated, as opposed to stationary phase when production of flagella is decreased and cells are non-motile (Edward Ruby, personal communication). The flagella during exponential phase may be disrupting the low shear environment thereby minimizing the effects of LSMMG on the cells and resulting in very few stress-associated genes being differentially regulated under the LSMMG environments at 12 h.

At 24 h, there was also expression of several other stress-associated genes (e.g. *blc*, *zwf*, *katA*), however, only *katA* has been previously described in *V. fischeri*. The *katA* gene encodes for a periplasmic catalase that is essential for the normal colonization of the host squid and is typically induced as cells approach stationary phase.^75^ The higher expression of *katA* during LSMMG in stationary phase suggests the cells are experiencing a more pronounced oxidative stress environment compared to the gravity controls. Similar results have been observed in several *Salmonella* spp., where bacteria grown under LSMMG conditions exhibited a higher resistance to hydrogen peroxide and increased catalase activity.^52^ Interestingly, although there was up-regulation of *blc*, which encodes for an outer membrane lipoprotein in *E. coli* expressed during osmotic stress,^72^ there were no observed significant DEGs associated with lipopolysaccharide biosynthetic genes or other cell membrane modifications, which have been reported to be differentially regulated under LSMMG.^18,22,47^ Together, the results reinforce the interpretation that the low shear environment of modeled microgravity does not significantly alter the transcriptional response *V. fischeri* cultivars, but that the few genes that are differentially expressed are primarily associated with environmental stress responses.

Although the transcriptome of WT cells did not display extensive changes in response to LSMMG, mutants defective in the global regulator Hfq exhibited a pronounced transcriptional response to LSMMG conditions (Fig. 1; Supplemental Table S1). The RNA-binding protein Hfq has been identified as an important transcriptional regulator in several pathogenic taxa in response to both spaceflight and microgravity analog environments.^33,34,47^ Additionally, the gene encoding Hfq has been shown to be down-regulated in several taxa, including *V. fischeri*, under LSMMG conditions.^23,47^ One of the major functions of Hfq is to bind to sRNAs, which then together subsequently target various mRNAs, thereby regulating or modulating the stability of the mRNAs.^93^ The sRNAs can also serve as activators or repressors of mRNA translation.^94^ During exponential phase, there was a significant accumulation of transcripts associated with the TCA cycle in the ∆*hfq* mutant. Up-regulation of genes associated with the TCA cycle has been observed under modeled microgravity^27,35,95^ and many TCA cycle genes are typically repressed by the Hfq-dependent sRNA RhyB,^77^ which recruits RNase E and facilitates mRNA degradation.^78^ In the ∆*hfq* mutant, the increase in recovered TCA cycle transcripts under LSMMG conditions likely reflects an inhibition of mRNA degradation rather than an up-regulation of these genes during LSMMG.

The ∆*hfq* mutants also exhibited an increase of transcripts associated with flagellar assembly under LSMMG conditions compared to WT cells during exponential phase growth (Fig. 3a; Supplemental Tables S1 and S2). The expression of flagellar assembly genes under microgravity conditions appears to be highly variable based on the taxa and whether the cells were exposed to actual spaceflight or analog conditions.^19,51,95,96^ The regulation of flagellar synthesis is complex and is not fully delineated in *V. fischeri*,^97^ although in many taxa it occurs both at the transcriptional and translational levels.^98^ In *E. coli* there are numerous Hfq-dependent sRNAs involved in the positive (e.g. McaS) and negative (e.g. ArcZ, OmrA, OmrB, SdsR, GadY, and OxyS) regulation of flagella synthesis, however, none of these Hfq-dependent sRNAs have been reported in the *V. fischeri* genome. The up-regulation of flagellar synthesis transcripts during exponential phase in LSMMG may suggest the cells are attempting to move out of potential zones of nutritional depletion. Alternatively, as there is a lack of differentially expressed flagellar synthesis transcripts during stationary phase when nutritional depletion is more severe, the results may simply suggest a lack of negative repression of the flagellar synthesis in the ∆*hfq* mutants during cell growth. A more detailed analysis of transcriptional and translational regulation of flagella synthesis in *V. fischeri* is needed.

In stationary phase growth, the ∆*hfq* mutants exhibited a pronounced increase in the expression of transcriptional regulators under both gravity-specific (e.g. Fis-family regulator, VF_1401; *cysB*), and LSMMG-specific (Cro/Ci family regulator VF_2037; *nrdR*) conditions. To our knowledge, none of these regulators have been reported to be differentially expressed during spaceflight or modeled microgravity conditions. For example, *nrdR* was first shown to positively regulate synthesis of ribonucleotide reductases in response to DNA damage and oxidative stress in *Streptococcus pyrogens*^99^ and more recently has been shown in *E. coli* to be involved in responding to iron starvation^100^ and the host immune system.^101^ As the stress responses of several taxa are altered under spaceflight and analog conditions,^95^ the role of these transcriptional regulators under microgravity-like stress conditions needs to be investigated further.

The ∆*hfq* mutant also exhibited significant DEGs associated with outer membrane proteins that are differentially expressed during stress conditions in a wide range of taxa. For example, *slp* is a carbon-starvation-induced gene that has been shown to be upregulated in *V. cholerae* ∆*hfq* mutants^102^ and is released in outer membrane vesicles.^103^ In *E. coli* the Slp lipoprotein is essential for acid and metabolic stress and is negatively regulated by the Hfq-dependent GvcB^104^. Although GvcB has not been reported in *V. fischeri* it has been reported in several environmental vibrios.^80^ These differential transcriptional responses in genes encoding for outer membrane proteins in the ∆*hfq* mutants may indicate that the outer membrane of the mutants may have a different composition compared to the WT cells under both gravity and LSMMG conditions. Recent studies have shown that *V. fischeri*-derived outer membrane vesicles can induce full developmental remodeling of the host light organ tissues,^105^ however, mutants defective in Hfq induce an altered phenotype including a decrease in the number of dying apoptotic cells in the host tissues under both LSMMG and gravity conditions.^23^ The mechanism for these decreased levels of apoptotic cells in ∆*hfq* mutants is not clear but may be the product of a remodeled outer membrane.

Although the full range of environmental factors that impact a host’s microbiome in the space environment has yet to be fully understood, this study provides insight into the role that microgravity may have on those beneficial microbes that typically associate with animal tissues. The results suggest that under normal growth conditions modeled microgravity does not negatively impact the transcriptional activities of *V. fischeri* indicating that the beneficial, mutualistic lifestyle of the bacterium is maintained under analog microgravity conditions. The results also deepen our understanding of the mechanisms by which organisms are adapting to changes in their nutritional environment and how the global regulator Hfq impacts the regulatory processes of *V. fischeri* in both LSMMG and gravity conditions. These results indicate that Hfq serves as an important mechanism by which *V. fischeri* regulates responses to external stimuli. As many of the mechanisms by which pathogenic and beneficial microbes sense and respond to their ever-changing environment are shared, it will be critical to continue to explore the processes by which microbes form complex communities and interact with their hosts during spaceflight to help mitigate any potential health threats during long-term missions.

## Methods

### Bacterial strains and growth conditions

The wild type strain *V. fischeri* ES114 (WT), which was isolated from an adult host squid *E. scolopes*^106^ was used as the parent strain for the deletion ∆*hfq* mutant and complementation (KV7142, ∆*hfq*; KV148, ∆*hfq* attTn7::ermR; KV149, ∆*hfq* attTn7::*hfq*; courtesy of K. Visick, Loyola University Chicago). The strains were grown aerobically overnight in seawater tryptone (SWT) agar at 28 °C, in which trace elements are at low concentration (e.g. phosphate (0.1 ppm)).^23^ High aspect ratio rotating vessels (HARVs; Synthecon, Houston, TX, USA) were used to model the microgravity environment as previously described.^49,62^ Briefly, each HARV was filled with 50 ml of SWT broth inoculated with *V. fischeri* culture at a concentration of 1 × 10^5^ cells per ml of SWT. The HARVs were either rotated around a horizontal axis to simulate microgravity (LSMMG) or a vertical axis to serve as a normal gravity (1 × *g*) control. The cultures were incubated in the HARVs at 12 and 24 h in the vertical LSMMG and horizontal gravity control positions at 23 °C to replicate temperatures cells would experience in the natural environment. The HARVs were rotated at a constant velocity of 13 rpm, which prevented *V. fischeri* cells from forming sedimentary aggregates and to match rotation speed used in comparable squid-vibrio experiments.^62^ Experiments were conducted in triplicate for each condition, strain, and time. Growth curves of all strains used in this experiment are visualized in Supplemental Fig. S1 and corresponded to previously published results.^23,62^ At the end of each HARV experiment *V. fischeri* were flash frozen in liquid nitrogen to halt gene expression and stored at −80 °C until RNA extraction.

### RNA extraction, cDNA synthesis, and sequencing

Each replicate *V. fischeri* WT and ∆*hfq* culture was thawed on ice and pelleted for RNA extraction. Total RNA was extracted in triplicate for each treatment using PowerSoil^®^ Total RNA Isolation Kit (Qiagen, Germantown, MD) according to manufacturer’s protocol and was treated with TURBO DNase (Thermo Fisher Scientific, Waltham, MA) to remove potential contaminating DNA. The RiboMinus rRNA removal kit (Thermo Fisher Scientific, Waltham, MA) was used to deplete large rRNAs and samples were processed with the Zymo RNA Clean & Concentrator kit (Zymo Research, Irvine, CA). The remaining mRNA was pooled between replicates, the concentration was determined by Qubit 2.0 (Thermo Fisher Scientific, Waltham, MA) and quality was evaluated with a 2100 Bioanalyzer generating RIN factor > 9 (Agilent Technologies, Santa Clara, CA). High-quality RNA was converted to cDNA using a modified SuperScript Double Stranded cDNA synthesis kit (Thermo Fisher Scientific, Waltham, MA). A total of three replicate cDNA libraries were generated for each treatment (note: only two libraries were generated for ∆*hfq* gravity controls) and sequenced using the Illumina NextSeq500 platform (2 × 150 bp paired-end reads; Illumina, San Diego, CA).

### Bioinformatic analysis

Sequences were quality trimmed and filtered with Trimmomatic v0.32 using default parameters.^107^ The quality of the output files was then analyzed using FastQC v0.10.1.^108^ Reads were then aligned to the *V. fischeri* ES114 reference genome (GenBank ID: ASM11800v1) using Bowtie 2 v2.2.8.^109^ Gene counts were obtained using HTSeq-count v 0.6.1.^110^ Genes with no expression across all conditions were removed. Differential expression analysis was conducted in R using the package DESeq2.^111^ Genes were considered significantly differentially expressed at adjusted *p*-value (padj) < 0.05. UpSetR was used to visualize the intersection of DEGs.^112^ The most recent KEGG database was accessed through the R package KEGGREST v1.16.1 to determine KEGG functional pathways and higher KEGG level classification.^113^ The top 103 DEGs among time treatment comparisons with one defined KEGG pathway were visualized in a heatmap. Expression values for heatmap were normalized with trimmed mean of *M*-values (TMM) using the NOISeq package^114^ and scaled by the sum of each row (*z*-score) using heatmap.2 in the ggplots package in R.^115^ Genes associated with multiple pathways at KEGG level 2, or had no specific KEGG pathway association, were not displayed in the heatmap.

### Real-time quantitative PCR (qRT-PCR)

Several significantly DEGs were chosen for targeted qRT-PCR confirmation. Primers are listed in Supplemental Table S4. The qRT-PCR reactions were prepared using the iTaq Universal SYBR Green One-Step Kit (Biorad, Hercules, CA) with 10 ng of RNA per reaction. Amplification and quantification were completed using a Biorad SCX9600 Real Time System (Biorad, Hercules, CA). The amplification conditions were as follows: an initial incubation at 50 °C for 10 min then 1 min at 95 °C followed by 39 cycles of 95 °C for 10 s and 60 °C for 15 s. Each comparison was run in triplicate and three technical replicates were run for each biological replicate. The relative expression of each gene was analyzed using the comparative Cq method (ΔΔCq) on the Biorad system. The gene *rpoD* was chosen as the housekeeping reference gene for normalization of transcript abundances as previously described.^116^

## Electronic supplementary material

Supplemental Material Main Document

Supplemental Tables Merged

## Data Availability

All reads have been deposited in the NASA GeneLab database as well as NCBI under Bioproject PRJNA357702.

## Electronic supplementary material

**Supplementary information** accompanies the paper on the *npj Microgravity* website (10.1038/s41526-018-0060-1).
