# Supplementary material for: Transcriptional profiling of the mutualistic bacterium Vibrio fischeri and an hfq mutant under modeled microgravity
Source: NPJ Microgravity. 2018 Dec 18;4:25. doi: 10.1038/s41526-018-0060-1 (PMC6299092; doi:10.1038/s41526-018-0060-1)
Supplement: Supplementary file 1 — Supplemental Material Main Document [file 41526_2018_60_MOESM1_ESM.pdf]

Supplemental Material:

Transcriptional profiling of the mutualistic bacterium *Vibrio fischeri* and an *hfq* mutant under modeled microgravity

Alexandrea A. Duscher[1], Ana Conesa[2], Mary Bishop[1], Madeline M. Vroom[1], Sergio D. Zubrizarreta[1], and Jamie S. Foster[1*]

Running Title: Impact of modeled microgravity on a mutualistic bacterium

[1]Department of Microbiology and Cell Science, University of Florida, Space Life Science Lab, Merritt Island, FL, 32953, USA

[2]Department of Microbiology and Cell Science, Institute of Food and Agricultural Research, Genetics Institute, University of Florida, Gainesville, FL, 32611, USA

*Corresponding Author:

Jamie S. Foster (jfoster@ufl.edu)

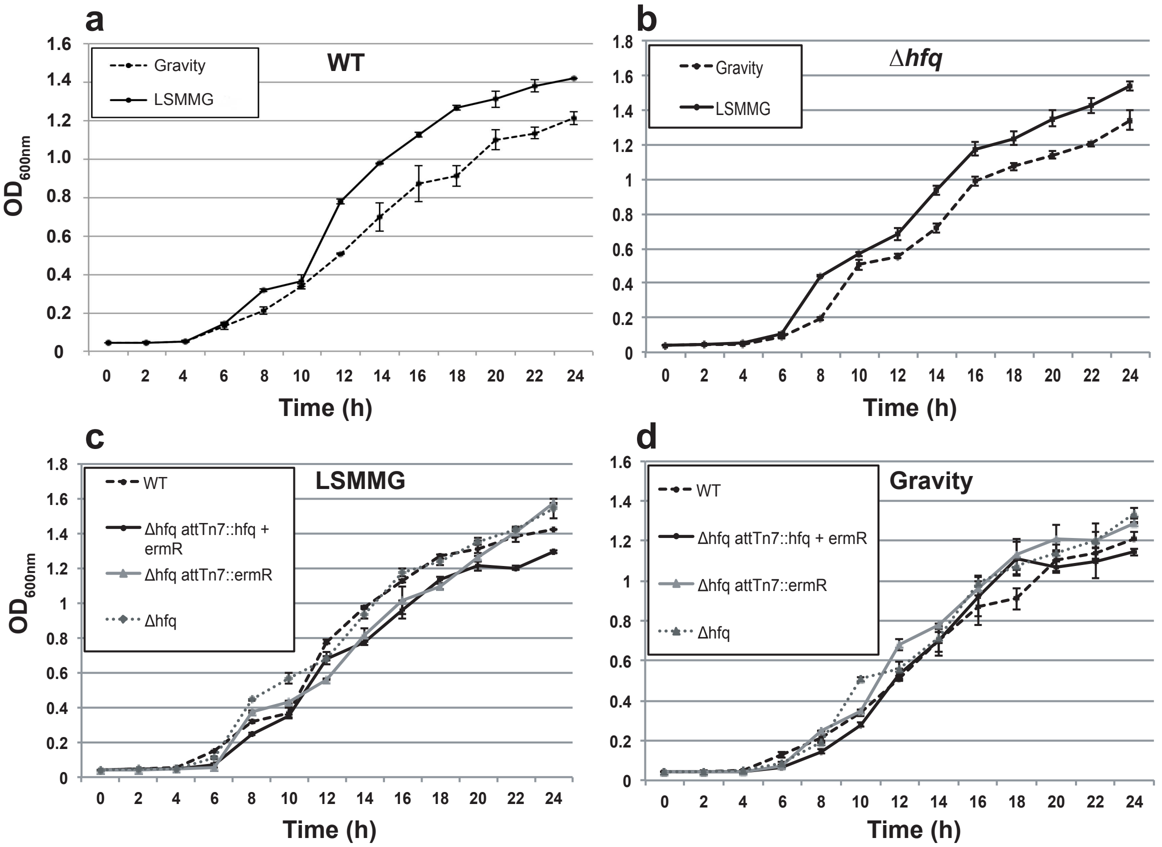

Supplemental Fig. S1. Growth curves of strains used in this study. (**a**) WT strain grown under gravity and LSMMG conditions. (**b**) Δ*hfq* mutant grown under gravity and LSMMG conditions. A comparison of all the strains including Δ*hfq* complementation mutants grown under LSMMG (**c**) and gravity (**d**) conditions.

Supplemental Table S1. Significant differentially expressed genes in pairwise comparisons at log$_2$-fold change of +/- 1 and padj ≤ 0.05. See excel sheet.

Supplemental Table S2. Normalized read counts of data associated with Figure 2. See excel sheet.

Supplemental Table S3. Significant differentially expressed genes within treatments at 12h compared to 24 h. See excel sheet.

Supplemental Table S4. Primers designed for qRT-PCR gene verification

Primer	Sequence (5'->3')	Product size	Tm (°C)
*rpoD*_F	AGCACGTACGATCCGTATTCC	121	60.0
*rpoD*_R	GCGTTCAGCAAGCTCTTCAG		59.8
*katA*_F	CCAGATAAGATGCTACAAGGTCG	146	58.7
*katA*_R	CCATCAACACGCATAGCACC		59.6
*flgK*_F	GCCGCACAAAGCTATGCAA	123	59.8
*flgK*_R	AGCAAACTGATTTGCAGCAGG		60.0
*flaA*_F	GAACCATCAATCGAAGGTGAGC	141	59.7
*flaA*_R	AACACCGATAGACACTTGTGC		58.6
*dnaK1*_F	GTGCGGTAACAATTCACGTACT	129	59.3
*dnaK1*_R	CGAATGTTACTTCGATTTGTGGC		58.9
*lpxD*_F	CGTGGTGCTATTGATGACACG	105	59.7
*lpxD*_R	ACCAGCCATTGCTGAACCA		59.9
